# Cone beam CT augmented fluoroscopy allows safe and efficient diagnosis of a difficult lung nodule

**DOI:** 10.1186/s12890-021-01697-y

**Published:** 2021-10-20

**Authors:** Roberto Piro, Matteo Fontana, Eleonora Casalini, Sofia Taddei, Marco Bertolini, Mauro Iori, Nicola Facciolongo

**Affiliations:** 1 Pulmonology Unit, Azienda Unità Sanitaria Locale - IRCCS di Reggio Emilia, Via Amendola 2, 42123 Reggio Emilia, Italy; 2 Medical Physics Unit, Azienda Unità Sanitaria Locale - IRCCS di Reggio Emilia, Reggio Emilia, Italy

**Keywords:** Cone beam CT, Bronchoscopy, Lung nodules, Interventional pulmonology

## Abstract

**Background:**

Detection of small peripheral lung nodules is constantly increasing with the development of low dose computed tomography lung cancer screening programs. A tissue diagnosis is often required to confirm malignity, with endobronchial biopsies being associated with a lower pneumothorax rate than percutaneous approaches. Endoscopic diagnosis of peripheral small size lung nodules is however often challenging using traditional bronchoscopy and endobronchial ultrasound alone. New virtual bronchoscopic navigation techniques such as electromagnetic navigational bronchoscopy (ENB) have developed to improve peripheral navigation, with diagnostic yield however remaining in the 30–50% range for small lesions. Recent studies have shown the benefits of combining Cone beam computed tomography (CBCT) with ENB to improve diagnostic yield to up to 83%. The use of ENB however remains limited by disposable cost, bronchus sign dependency and inaccuracies due to CT to body divergence.

**Case presentation:**

This case report highlights the feasibility and usefulness of CBCT-guided bronchoscopy for the sampling of lung nodules difficult to reach through traditional bronchoscopy because of nodule size and peripheral position. Procedure was scheduled in a mobile robotic hybrid operating room with patient under general anaesthesia. CBCT acquisition was performed to localize the target lesion and plan the best path to reach it into bronchial tree. A dedicated software was used to segment the lesion and the bronchial path which 3D outlines were automatically fused in real time on the fluoroscopic images to augment live guidance. Navigation to the lesion was guided with bronchoscopy and augmented fluoroscopy alone. Before the sampling, CBCT imaging was repeated to confirm the proper position of the instrument into the lesion. Four transbronchial needle aspirations (TBNA) were performed and the tissue analysis showed a primary lung adenocarcinoma.

**Conclusions:**

CBCT and augmented fluoroscopy technique is a safe and effective and has potential to improve early stage peripheral lesions endobronchial diagnostic yield without ENB. Additional studies are warranted to confirm its safety, efficacy and technical benefits, both for diagnosis of oncological and non-oncological disease and for endobronchial treatment of inoperable patients.

## Background

Lung cancer is the most common cause of cancer death worldwide with 2.21 million new cases and 1.8 million cancer deaths in 2020 [1]. Early diagnosis is a clinically important challenge to improve prognosis. The development of low-dose chest computed tomography (CT) lung screening programs has resulted in an increased incidence of small nodules suspected of early-stage lung cancer requiring sampling to confirm malignancy [2, 3]. Percutaneous needle aspiration has been associated with a high sensitivity (up to 90%) [4, 5] but is limited by a significant complication rate with up to 25% pneumothorax reported in the literature [6]. Conventional fluoroscopy-guided transbronchial biopsies are associated with significantly lower pneumothorax rates but also lower diagnostic yield, in particular for early stage small peripheral nodules [7]. In the last years, new interventional pulmonology technologies have been to obtain safe and effective tissue collection [8, 9] by improving navigation guidance through the bronchial pathway, device flexibility to reach lesions at tight angulation relative to the airway, and real time assessment of the relationship between the sampling device and the target lesion which can be even smaller than 1 cm. To improve navigation and lesion reach, thin/ultrathin bronchoscopes [10], preoperative CT-based virtual bronchoscopic navigation (VBN) [11, 12] such as electromagnetic navigational bronchoscopy (ENB) [13], bronchoscopic transparenchymal nodule access (BTPNA) [14], transbronchial access tools (TBAT) [15] and robotic-assisted bronchoscopy [16–18] are the main advances now available. Radial probe endobronchial ultrasound (R-EBUS) is a useful tool to study a lesion, when correctly reached [19]. ENB has significantly improved peripheral endobronchial navigation, but remains limited by consumable cost, bronchus sign dependency and preoperative CT-to body divergence resulting in a relatively low [30–73%] diagnostic yield in particular in small lesions [20–22]. The use of cone beam CT (CBCT) in combination with ENB has been described to confirm proper device position within the lesion before sampling, significantly increasing diagnostic yield to [70–83%] [23–25].

CBCT is an intraoperative 3D imaging technique developed in the early 2000s and adopted as standard of care in many endovascular and percutaneous procedures to improve targeting, treatment planning and assessment [26, 27]. In modern interventional radiology and hybrid operating rooms, CBCT-based 3D advanced procedural planning is fused on fluoroscopy to augment live guidance. CBCT and augmented fluoroscopy technical and clinical benefits have been demonstrated and their use is established in interventional radiology, interventional oncology and minimally invasive vascular surgery procedures. These technologies have recently been applied also in the field of interventional pulmonology [27–29], with or without VBN [11, 23, 30–32], and in thoracic surgery to streamline video-assisted resections [33].

## Case presentation

**Fig. 1:**
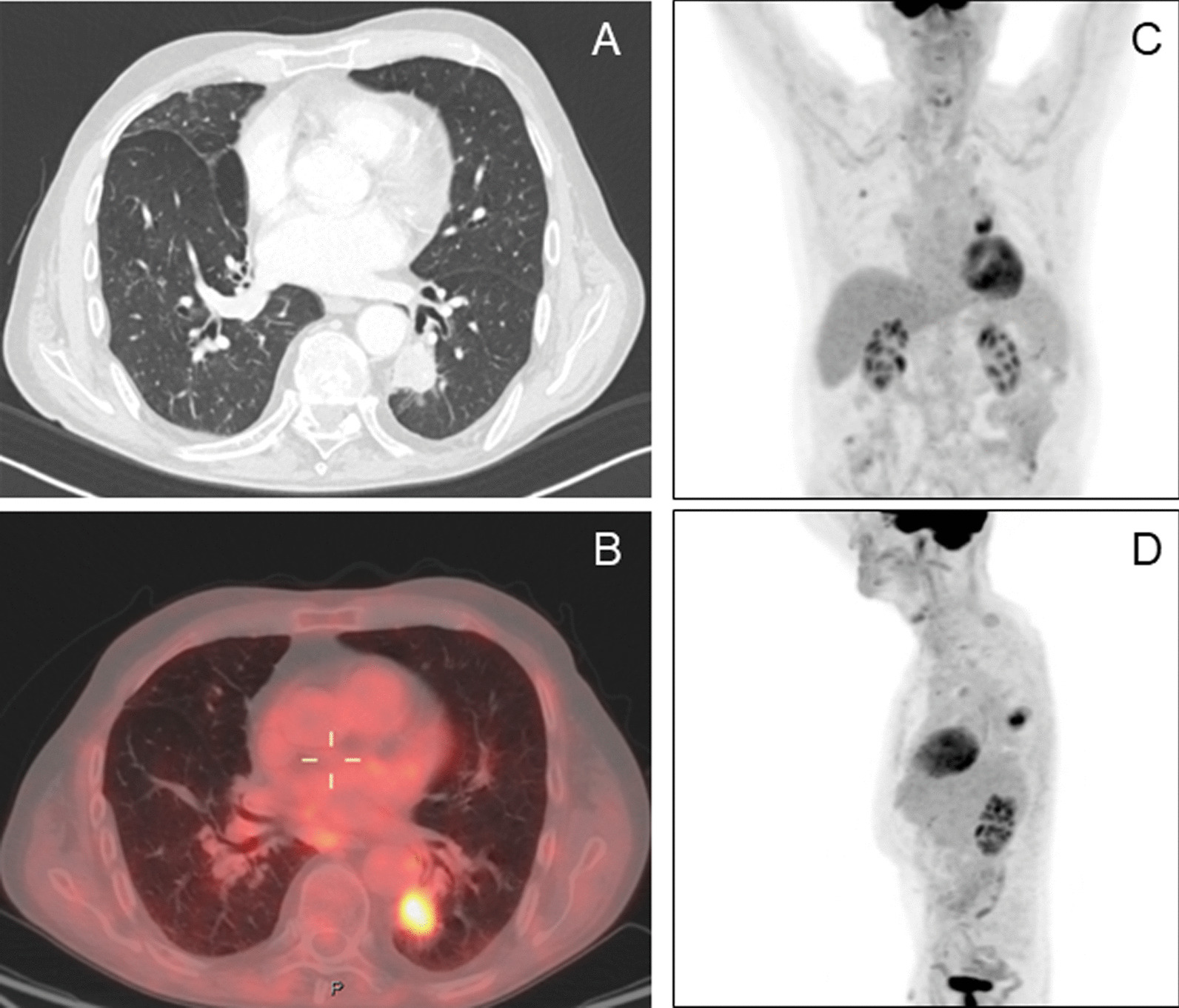
Chest CT scan and FDG PET-CT. **A** Chest CT scan in axial plane. **B** FDG PET-CT in coronal plane. **C** FDG PET-CT in sagittal plane. **D** Chest CT scan and FDG PET-CT in axial plane

**Fig. 2:**
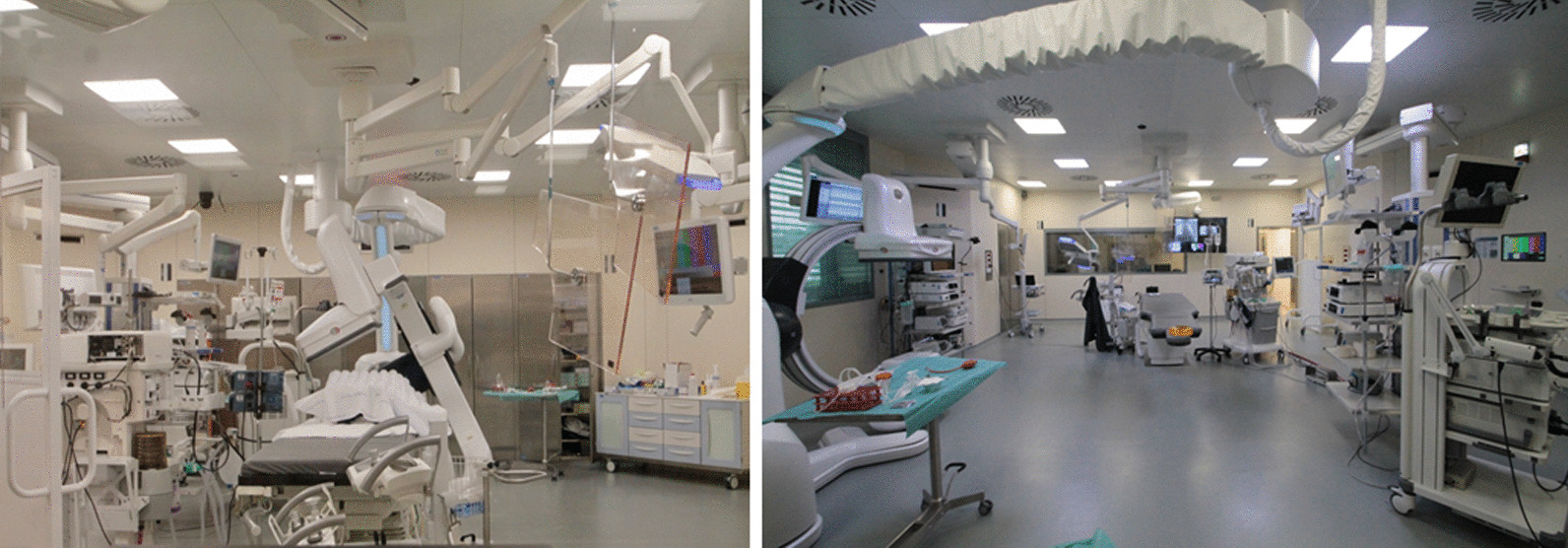
The hybrid operating theatre. The hybrid operating theatre with the instrumentation for the CBCT-guided bronchoscopy

A 78-year-old former smoker (80 p/y) male patient was admitted for a 37 * 28 mm left lower lobe pulmonary nodule detected on follow up CT scan. Patient history included a right upper lobe lung resection for adenocarcinoma in 2008 and a trans-urethral resection for prostate adenocarcinoma in 2019 (Gleason Score 6 3 + 3; Grade Group 1); his medical history was also significant for chronic obstructive pulmonary disease (COPD) with frequent exacerbations and for a hypokinetic cardiomyopathy. Nodule had increased in size compared to a previous CT scan and it presented a significant uptake (SUVmax = 6.4) at 18 FDG-PET/CT (Fig. 1). No suspicious mediastinal lymphadenopathy was detected on both chest CT scan and FDG-PET/CT. The lung lesion was difficult to reach through traditional bronchoscopy combined with standard C-arm fluoroscopy guidance due to the nodule’s position and size. A percutaneous transthoracic needle aspiration was judged as a high-risk procedure based on the nodule position and the risk of pneumothorax in a COPD patient. Considering the diagnostic difficulty, a CBCT-guided bronchoscopy was proposed and accepted by the patient. Procedure was scheduled in a mobile robotic hybrid operating room (Fig. 2; Discovery IGS 740, GE Healthcare, Chicago, IL) providing intraprocedural 3D and superior 2D imaging compared to standard surgical C-arms, along with advanced planning, guidance and assessment with augmented fluoroscopy. The procedure was conducted through a laryngeal mask in general anaesthesia. A standard flexible video bronchoscope with a 2.0 mm working channel was used (BF-H190, Olympus Medical Systems, Tokyo, Japan).

**Fig. 3:**
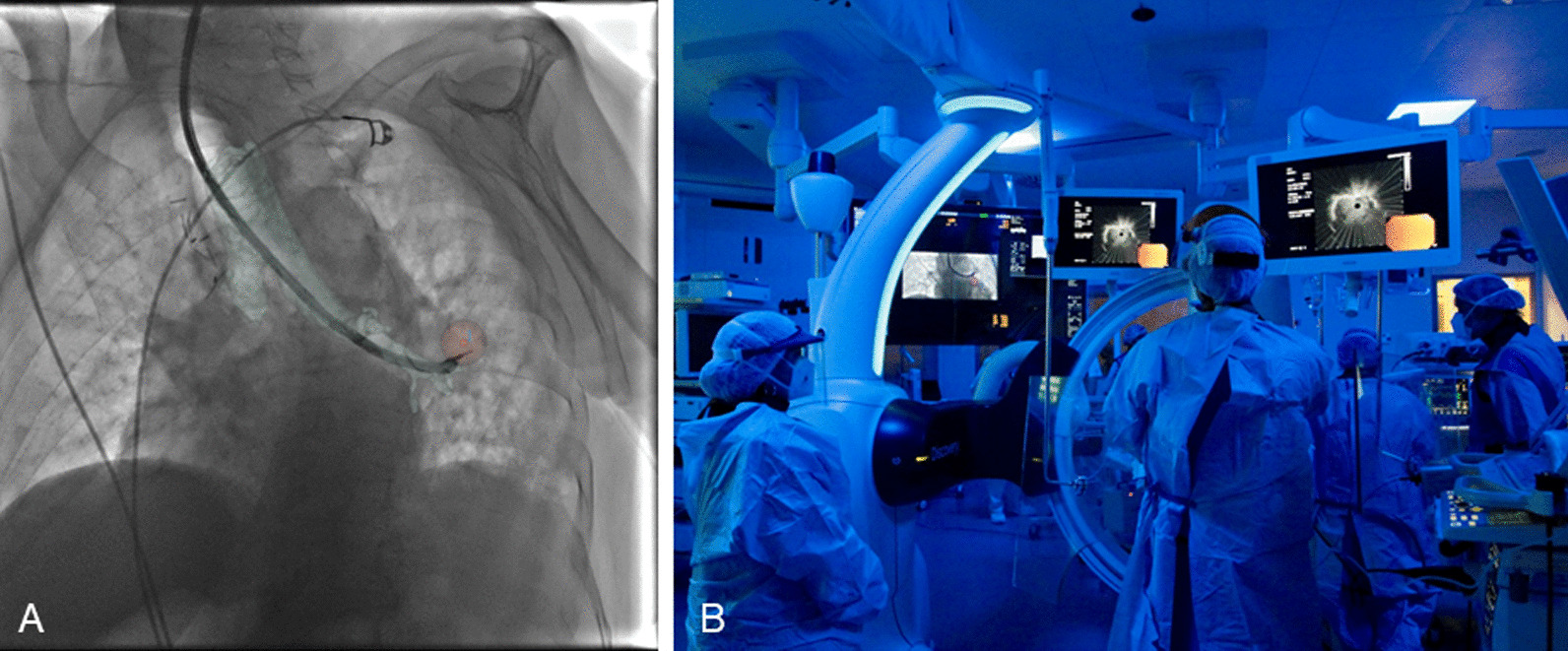
CBCT combined with R-EBUS. **A** The tumor (red) and the endobronchial path (blue) CBCT-based 3D volumes are automatically fused on the two-dimensional x-ray fluoroscopy imaging to augment live guidance. **B** Augmented fluoroscopy in combination with R-EBUS and bronchoscopic images

Thorax CBCT imaging was initially acquired to define target position and the easiest path to reach it within the bronchial tree. Images were obtained through a 5 s rotational acquisition around the patient, providing intraprocedural CT-like soft tissue cross-sectional imaging of the lungs. To minimize CBCT reconstruction blurring effects due to breathing, patient was put into apnoea during the acquisition. Using dedicated CBCT augmented planning and guidance software (ASSIST, GE Healthcare), interventional pulmonologist segmented the volumes of interest constituted by the target to be biopsied and the optimal endobronchial path to reach it. Tumor and endobronchial path CBCT-based 3D volumes were then automatically fused on the two-dimensional x-ray fluoroscopy imaging to augment live guidance (Fig. 3A). Fusion remained automatically registered following table movements and C-arm angulations. Fusion opacity and rendering options (outline, volume) were adjustable from table side to maximize live fluoroscopy and augmented guidance visualization along the procedure. Augmented fluoroscopy was used in combination with traditional bronchoscopy to guide the navigation following the planned path (Fig. 3B).

A R-EBUS probe (UM-S20-17S; Olympus Medical Systems) helped to identify the correct fifth generation bronchus leading to the lesion; it was a branch of the segmental LB6 (apical of the left lower bronchus). Then a needle was put in that pathway and a second CBCT acquisition was performed to confirm that the tip was into the target lesion. Tissue sampling was performed under augmented fluoroscopy guidance with four transbronchial needle aspirations (TBNA) in the target site. The angiographic unit was optimised in terms of quality imaging level and dose to the patient [34]. Total fluoroscopy time was 13.4 min. Total kerma area product (KAP) and air kerma at the interventional reference point (IRP) were 59.15 Gy cm^2^ and 159.3 mGy, respectively. Regarding these data, fluoroscopy accounts for 22.13 Gy cm^2^ and 69.1 mGy, while CBCT explains for 37.02 Gy cm^2^ and 90.2 mGy, respectively. The patient’s peak skin dose was 148 mGy. The exposure to ionising radiation for the first operator, the closest to the radiation field, was 2 µSv (body dose equivalent), 1.1 µSv (lens dose equivalent), and 5.7 µSv (hand dose equivalent) [35, 36]. These doses were estimated using the measurements carried out around the angiograph and assuming the worst-case scenario, i.e., without considering the attenuation of the operator protection devices (shielded aprons and collars of 0.5 mm of Pb equivalent). These dose estimates agreed with the operator dosimeters whose readings were not distinguishable from their minimum detection threshold [10 µSv, measurements in Hp (10) and Hp (0.07) [35]]. Rapid on-site evaluation (ROSE) was not performed. The procedure ended without any complication. The tissue sampling showed epithelial cells consistent with adenocarcinoma (TTF1 positive, p40 negative), confirming the pulmonary primitivity of the neoplasia. The patient was referred to the oncologist for appropriate treatment.

## Discussion and conclusions

Transbronchial sampling of small peripheral lung lesions is increasingly important [7, 37]. Virtual navigation tools have been developed to improve endobronchial planning and guidance towards the target or the region of interest to be investigated, but they are limited by consumable cost and inaccuracies between the preoperative CT and the live patient, while lacking real-time intraoperative 3D imaging of the device/target spatial relationship before sampling. CBCT and augmented fluoroscopy are not yet a widespread approach for endobronchial biopsy of small peripheral pulmonary nodules but they could be a promising avenue for simplified and more accurate navigation and for real-time assessment. Indeed, especially when used together with R-EBUS, they allow a target confirmation and a precise identification of the relationship between the airways and the lesion. CBCT advanced guidance delayed adoption in interventional pulmonology is probably due to preliminary clinical experiences describing complications (pneumothorax, COPD exacerbation) and high radiation exposure levels [25, 29, 38]. Large clinical trials on the accuracy and safety of this technique are lacking.

We herein report the case of a fragile patient with a difficult to reach lung nodule, successfully sampled through endobronchial approach using CBCT and augmented fluoroscopy as main guidance. First, this case highlights and confirms the safety of bronchoscopy in elderly patients with multiple comorbidities [39]. Then, it provides evidence on the feasibility and the usefulness of CBCT advanced guidance in the sampling of difficult lung nodules. In this case it is also important to highlight the use of TBNA, a tool that has a high diagnostic accuracy for peripheral pulmonary lesions with and without the presence of “bronchus sign” on CT [39, 40]; on the other hand, it allowed us to reach a more peripheral site during the exam. CBCT seamless multimodality integration in the latest interventional suites, allowing to combine information from different imaging modalities (CBCT, CT, PET/CT, MRI) is also of interest and will be assessed in our practice. Additional studies are warranted to confirm the safety and efficacy of this technique, opening the avenue of CBCT advanced imaging to several pulmonary clinical applications such as non-oncological diseases diagnosis [41, 42], endobronchial treatment for inoperable patients (cryotherapy, photodynamic therapy, microwave ablation, radiofrequency ablation or, thermal vapor ablation) [43, 44] and for the placement of fiducial markers [45].

## Data Availability

The datasets used and analyzed during the current study are available from the corresponding author on reasonable request.

## Abbreviations

3D: Three-dimensional space
CBCT: Cone beam computed tomography
COPD: Chronic obstructive pulmonary disease
CT: Computed tomography
ENB: Electromagnetic navigational bronchoscopy
FDG: Fluorodeoxyglucose
MRI: Magnetic resonance imaging
PET: Positron emission tomography
R-EBUS: Radial probe endobronchial ultrasound
SUVmax: Maximum standardized uptake value
TTF1: Transcription termination factor 1
TBNA: Transbronchial needle aspiration
VBN: Virtual bronchoscopic navigation
